# Prader-Willi syndrome: Methylation study or fluorescence *in situ* hybridization first?

**DOI:** 10.4103/0971-6866.73417

**Published:** 2010

**Authors:** Khalil Hamzi, Afaf Ben Itto, Sanaa Nassereddine, Sellama Nadifi

**Affiliations:** Laboratory of Human Genetics, Faculty of Medicine, Casablanca, Morocco; 1 Department of Pediatrics, University Hospital, Casablanca, Morocco

**Keywords:** Fluorescence *in situ* hybridization, methylation, obesity, Prader–Willi syndrome

## Abstract

Prader–Willi syndrome (PWS) is neurogenetic disorder involving the imprinting mechanism at 15q11–13 region. We report a 4-year-old girl who was referred to our laboratory to be investigated for clinical obesity, mental deficiency and respiratory problems. The patient was born for non-consanguineous and healthy biological parents. After normal pregnancy, the patient was delivered by cesarean section at full term, with a birth weight of 2500 g, and the height and head circumference were unknown. In neonatal stage, she presented severe hypotonia with feeding problems. Her developmental progress was delayed. She walked and developed speech at the age of 3 years. Since the age of 3 years, she presented severe dental problems. Methylation study had confirmed the diagnosis, and for detecting etiology, fluorescence *in situ* hybridization using probes for small nuclear ribonucleoprotein polypeptide N (SNRPN), which map inside the chromosomal region 15q11–15q13, was necessary to confirm the 15q11–15q13 deletion of paternal chromosome 15, which is the predominant genetic defect in PWS. In conclusion, we report this case with an objective to reinforce the necessity of analysis of DNA methylation within the 15q11–13 region, which is an important tool for the correct diagnosis among children presenting with neonatal hypotonia, mental deficiency and obesity.

## Introduction

Prader–Willi syndrome (PWS; OMIM 176270) is one of the most common genetic disorders involving non-Mendelian inheritance in the form of genomic imprinting. It occurs with an incidence of 1 in every 15,000 live births and is characterized by severe hypotonia and feeding difficulties in early infancy, followed in later infancy or early childhood by excessive eating and gradual development of morbid obesity. Approximately 70% of individuals with PWS have a 15q11.2–q13 deletion on the paternally inherited chromosome 15, whereas 25% have maternal uniparental disomy (UPD), 5% have an imprinting center sequence variant and 1% of the patients have a structural chromosome rearrangement involving 15q11.2–q13. Regardless of the etiology, PWS patients obtain only the methylated allele(s) in the promoter region of the small nuclear ribonucleoprotein polypeptide N (*SNRPN*) gene.

## Case Report

**Figure 1:**
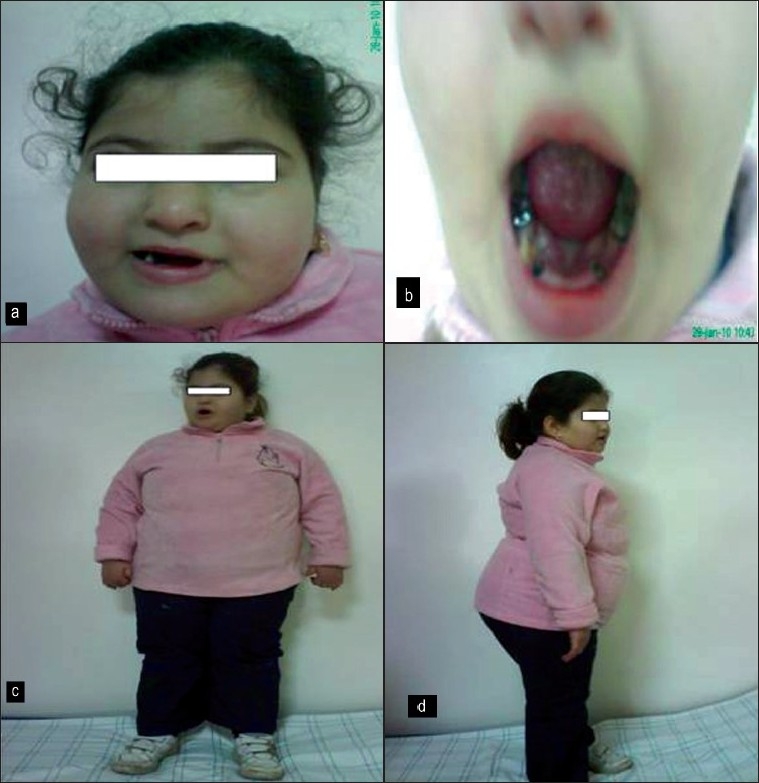
(a) Show the typical dysmorphic features of Prader Willi. (b) Severe dental problems. (c) Figures showing the severity of obesity (50 kgs for 4 years old). (d) Figures showing the severity of obesity (50 kgs for 4 years old).

We report a 4-year-old girl who was referred to our laboratory to be investigated for clinical obesity, mental deficiency and respiratory problems. The patient was born for non-consanguineous and healthy biological parents. After normal pregnancy, the patient was delivered by cesarean section at full term, with a birth weight of 2500 g; height and head circumference were unknown. In neonatal stage, she presented severe hypotonia with feeding problems. Her developmental progress was delayed. She walked and developed speech when she was 3 years old. When she was examined at the age of 3 years, she presented severe dental problems, mental retardation and a weight of 50 kg for 100 cm [Figure 1].

**Figure 2:**
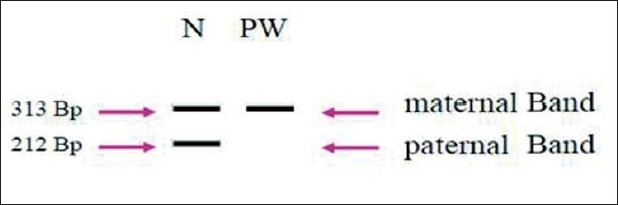
PCR Profil obtained with MS-PCR (N=Normal, PW = Prader Willi).

Methylation study using allele specific primers has confirmed the diagnosis of PWS showing the absence of the non-methylated paternal copy of *SNRPN* [Figure 2].

## Discussion

**Figure 3:**
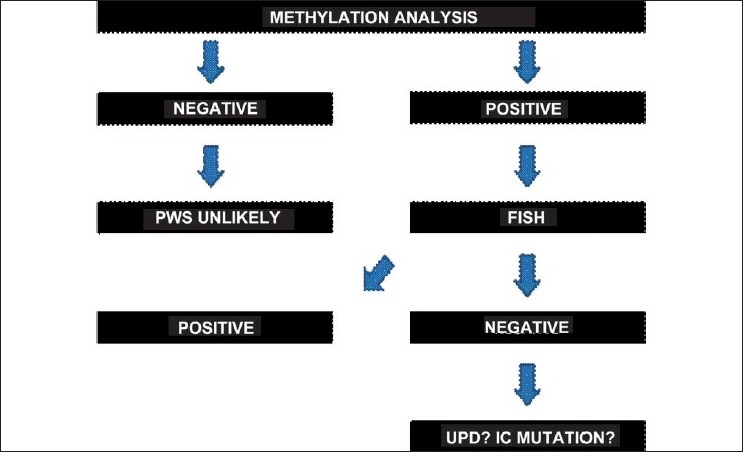
Diagnosis strategy of Prader-Willi syndrome.

Methylation analysis by Southern blotting using SNRPN probe detects over 99% of subjects with PWS.[1] The SNRPN probe has been validated as a diagnostic test for PWS.[2] Few subjects have been reported in the literature with typical PWS features and normal methylation studies.[3] Actually, several methods involving polymerase chain reaction (PCR) analysis for the molecular diagnosis of PWS have been developed, including methylation specific PCR (MS-PCR), fluorescence melting curve analysis (MS-HRMA) and reverse transcription PCR (RT-PCR).[4] These techniques have several advantages over Southern blot analysis for the diagnosis of PWS, including a rapid turnaround time and a smaller amount of DNA template required. As with Southern blot analysis, each method detects virtually all cases of PWS but they do not give any information on the etiology of the PWS.[4] Approximately 70% of patients with PWS carry microdeletions and most of the remaining PWS patients were later found to have UPD for chromosome 15.[5] The etiologic heterogeneity of PWS makes it difficult to establish uniform diagnostic methods that would detect most positive cases. Fluorescence *in situ* hybridization (FISH) analysis to identify the large microdeletions on the chromosome 15q is available, but only 70% of PWS cases are identified.[6] The diagnostic approaches that the different laboratories use differ from each other. Some laboratories begin their examinations with FISH to detect deletions, followed by the DNA methylation test, if the deletion is not found. However, beginning with the DNA methylation test, deletions as well as uniparental disomy and imprinting defects can be recognized.[7] In the case of an abnormal methylation imprint, FISH and microsatellite analysis are recommended to distinguish between microdeletion and uniparental disomy [Figure 3].[8]
